# Real-World Treatment Patterns and Sequencing of ALK-Inhibitors in ALK-Positive NSCLC Treated with Palliative Intent

**DOI:** 10.3390/cancers18213470

**Published:** 2026-10-27

**Authors:** Leyla Ay, Julie Krainer-Jacobs, Anna Lang-Stöberl, Vania Mikaela Rodriguez, Hannah Fabikan, Christoph Weinlinger, Maximillian Johannes Hochmair, Nino Müser, Robab Breyer-Kohansal, Marie-Kathrin Breyer, Arschang Valipour

**Affiliations:** 1 Karl Landsteiner Institute of Lung Research and Pulmonary Oncology and Department of Respiratory and Critical Care Medicine, Clinic Floridsdorf, Vienna Healthcare Group, 1210 Vienna, Austria; 2 Karl Landsteiner Institute of Lung Research and Pulmonary Oncology, 1210 Vienna, Austria; 3 Karl Landsteiner Institute of Lung Research and Pulmonary Oncology and 2(nd) Department of Internal Medicine with Pulmonology, Clinic Ottakring, Vienna Healthcare Group, 1160 Vienna, Austria; 4 Ludwig Boltzmann Institute for Lung Health and Department of Respiratory and Pulmonary Diseases, Clinic Hietzing, Vienna Healthcare Group, 1130 Vienna, Austria; 5 Ludwig Boltzmann Institute for Lung Health and Department of Respiratory and Pulmonary Diseases, Site Penzing of Clinic Ottakring, Vienna Healthcare Group, 1140 Vienna, Austria

**Keywords:** ALK inhibitors, therapeutic sequencing, NSCLC, multicenter registry, real-world data

## Abstract

Background: ALK-rearranged non-small cell lung cancer (NSCLC) is a molecularly defined subtype that is highly sensitive to ALK tyrosine kinase inhibitors (ALK inhibitors). With multiple generations of ALK inhibitors available, real-world data on treatment patterns and sequencing in routine clinical practice remain limited. Methods: This retrospective multicenter registry analysis included patients with ALK-positive NSCLC treated with palliative intent and enrolled in the LALUCA registry between November 2020 and September 2025 at four lung cancer centers in Vienna. ALK alterations were confirmed by next-generation sequencing. Demographic, clinical, and treatment-related data were analyzed descriptively. Results: Twenty-eight patients were included (median age, 54 years [range, 26–84]; 46.4% female). Most patients had stage IV disease (85.7%) and adenocarcinoma histology (92.9%). Overall, 49 systemic treatment lines were administered, including 41 ALK inhibitor treatment lines (22 second-generation and 19 third-generation). At data cut-off, 39.0% of ALK inhibitor treatment lines were ongoing, with a higher proportion of ongoing treatment observed among third-generation compared with second-generation ALK inhibitors (52.6% vs. 27.3%). The Kaplan–Meier-estimated median treatment duration was 287 days (95% CI, 172-NE) for second-generation and 736 days (95% CI, 376–NE) for third-generation ALK inhibitors. Objective response rates were 77.3% and 73.7%, respectively. Adverse events of any grade occurred in 73.2% of treatment lines, while grade ≥ 3 adverse events occurred in 12.2%. Dose reductions and temporary treatment interruptions were used to manage toxicity and allowed for treatment continuation in most affected treatment lines. Conclusions: This multicenter real-world analysis provides insights into contemporary ALK inhibitor treatment patterns and sequencing in routine clinical practice. Third-generation ALK inhibitors were associated with longer observed treatment durations in this cohort, although these findings should be interpreted cautiously given the small sample size, retrospective analysis, and potential differences in treatment selection and follow-up. Overall, toxicity was generally manageable, with dose modifications and temporary treatment interruptions frequently allowing for continuation of ALK inhibitor therapy.

## Simple Summary

Lung cancer with changes in the anaplastic lymphoma kinase (ALK) gene is a rare subtype that can be effectively treated with targeted medicines called ALK inhibitors. Several generations of these drugs are now available, but there is limited information on how they are used in everyday clinical practice outside of clinical trials. In this multicenter real-world study, we analyzed treatment patterns in patients with advanced ALK-positive non-small cell lung cancer treated at four specialized centers in Vienna. We evaluated how different ALK inhibitors were used over multiple lines of therapy, how long patients remained on treatment, and how side effects were managed. Our findings show increasing use of newer ALK inhibitors, prolonged treatment exposure, and effective management of side effects through dose adjustments and temporary treatment interruptions. These results provide valuable real-world evidence that may help optimize treatment sequencing and support clinical decision-making for patients with ALK-positive lung cancer.

## 1. Introduction

Anaplastic lymphoma kinase (ALK) gene rearrangements define a distinct molecular subgroup of non-small cell lung cancer (NSCLC), occurring in approximately 3–5% of patients with advanced disease and are predominantly found in younger patients with minimal or no smoking history and adenocarcinoma histology [1,2]. ALK rearrangements result in constitutive kinase activity that drives tumorgenesis, making them highly actionable therapeutic targets [1,3].

The introduction of ALK-targeted tyrosine kinase inhibitors (ALK inhibitors, ALK inhibitor) has transformed the clinical management of ALK-positive NSCLC. The first-generation ALK inhibitor, crizotinib, demonstrated superior efficacy compared with platinum-based chemotherapy and established targeted therapy as the new standard of care in ALK-rearranged disease [4]. Subsequent generations of ALK inhibitors—such as alectinib, brigatinib and lorlatinib—have shown improved potency, better central nervous system penetration, and activity against resistance mutations, yielding significant improvements in progression-free survival and intracranial control in the first-line setting [5,6,7,8,9]. Accurate molecular characterization is essential for identifying patients with ALK-rearranged NSCLC who may benefit from ALK-targeted therapy [10].

Despite these advances, disease progression remains inevitable for most patients, underscoring the need for sequential treatment strategies that maximize the benefit of multiple lines of ALK inhibitor therapy. Real-world evidence is particularly important because it captures the efficacy–effectiveness gap observed between clinical trials and routine clinical practice [11]. In ALK-positive NSCLC, real-world studies have demonstrated heterogeneous treatment sequences and provided insights into the effectiveness, tolerability, and clinical outcomes associated with sequential ALK inhibitor therapy [12,13,14]. However, comprehensive real-world analyses describing first-line ALK inhibitor selection and subsequent therapeutic pathways remain limited.

To address this gap in knowledge, we analyzed treatment patterns in patients with ALK-positive NSCLC treated with palliative intent in clinical practice between November 2020 and September 2025. Emphasis was placed on the generation of an ALK inhibitor selected for first-line therapy and on subsequent treatment lines to characterize real-world therapeutic sequencing in this molecularly defined subgroup.

## 2. Material and Methods

This study was conducted within the framework of the observational LALUCA registry (ClinicalTrials.gov identifier: NCT04733430) [15]. LALUCA includes lung cancer patients at the time of diagnosis and follows their course of disease capturing real-world data on clinical characteristics, diagnostic pathways, treatment patterns, and outcomes of unselected patients with lung cancer in routine practice, as previously described [15]. Briefly, adult patients (≥18 years) with newly diagnosed lung cancer of any histological subtype or stage are enrolled at four lung cancer institutions in Vienna after providing written informed consent. For deceased patients, the requirement for consent was waived by the ethics committee. Patients are prospectively followed at six-month intervals until death or loss to follow-up. Recruitment commenced in November 2020 and remains ongoing. The present analysis focuses on patients with ALK-rearranged lung cancer treated with palliative intent and aims to describe treatment patterns and therapeutic sequencing in this molecularly defined subgroup. Particular emphasis was placed on the selection of different generations of anaplastic lymphoma kinase inhibitors (ALK inhibitors) in the first-line setting and across subsequent lines of therapy. We included all eligible patients enrolled between 1 November 2020 and 30 September 2025 at the participating centers. Patients were required to have a confirmed ALK rearrangement detected by next-generation sequencing (NGS) and to have received systemic therapy with palliative intent. Demographic characteristics, tumor features, and treatment selection across all therapy lines were analyzed. In a small number of cases, patients who had initiated treatment before registry enrollment were included, and pre-enrollment clinical data were retrospectively collected from medical records. Consequently, treatment initiation dates may precede the start of patient recruitment.

### 2.1. Study Objectives

The primary objective of this analysis was to characterize real-world treatment patterns and therapeutic sequencing in patients with ALK-positive lung cancer receiving palliative systemic therapy within the LALUCA registry.

Secondary objectives were to assess the distribution of different generations of ALK inhibitors used in the first-line setting and across subsequent lines of therapy, and to describe patient and tumor characteristics associated with treatment selection. In addition, treatment sequences over the disease course were analyzed to reflect real-world therapeutic pathways.

### 2.2. Statistical Analyses

Due to the low prevalence of ALK rearrangements of 3–5% in NSCLC patients, the study was designed as a retrospective analysis of registry data and relied on descriptive statistics only. The sample was not intended or powered for inferential analyses; thus, descriptive measures were considered the most appropriate means to summarize observed patterns and to characterize this small study population.

Outcomes of interest were the descriptive assessment of patient, tumor and treatment characteristics, and the median duration of treatment (DOT), as well as an efficacy and safety analysis assessing the objective response rate (ORR), progression-free survival (PFS) and adverse events and treatment reduction including time to dose reduction (TTDR) and treatment holiday including time to treatment holiday (TTH) and respective reasons.

Patient, tumor and treatment characteristics are presented descriptively and are expressed as frequencies and proportions for categorical data and as the mean or median (as appropriate) plus the range for continuous data. The DOT was defined as the time between the first and last administered treatment for each line of therapy and was estimated using the Kaplan–Meier method. Patients remaining on treatment at the data cut-off date (30 June 2026) were censored at data cut-off. For descriptive purposes, observed treatment duration among patients who discontinued treatment was additionally summarized as the median and range. The objective response rate (ORR) was defined as the proportion of subjects who achieved either partial and complete response (PR/CR) as the best overall response (BOR) according to the RECIST 1.1 criteria. Responses classified as not evaluable (NE) or unknown were considered non-responses. The median progression-free survival (PFS) was defined as the time from the start of therapy until disease progression or death for all patients. Patients who were alive and progression-free at the time of the last follow-up were censored at the date of their last clinical assessment. Overall survival (OS) was defined as the time from diagnosis to death from any cause. Survival probabilities were estimated using the Kaplan–Meier method, with patients who were alive at the last follow-up censored at the date of their last contact. The median TTDR was defined as the time from the start of therapy to treatment reduction and the median TTH was defined as the time from the start of therapy to treatment holiday.

For temporal analyses of choice of treatment, patients were grouped according to treatment initiation during 2019–2021 versus 2022 and onward. This categorization was chosen to reflect a relevant change in clinical practice following the approval of lorlatinib for first-line treatment of ALK-positive advanced NSCLC in 2022 and its subsequent implementation in routine care in Austria from 2022 onwards.

This analysis employed exclusively descriptive statistical methods and no inferential statistical models or adjusted analyses were performed. The findings are intended solely to summarize patient characteristics, treatment courses, and observed treatment duration without implying causal relationships or providing hypothesis-driven comparisons beyond simple group descriptions. Consequently, subgroup comparisons should be interpreted carefully, as the limited number of patients and the exploratory design of the study do not allow for the detection of statistically robust differences. All data were analyzed with R 4.4.1.

## 3. Results

### 3.1. Patient Characteristics

Within the LALUCA registry, an unselected population of patients with lung cancer is enrolled across participating centers in Vienna, with approximately 400 new patients included per year and more than 2778 patients enrolled at analysis enrollment date [15].

**Figure 1:**
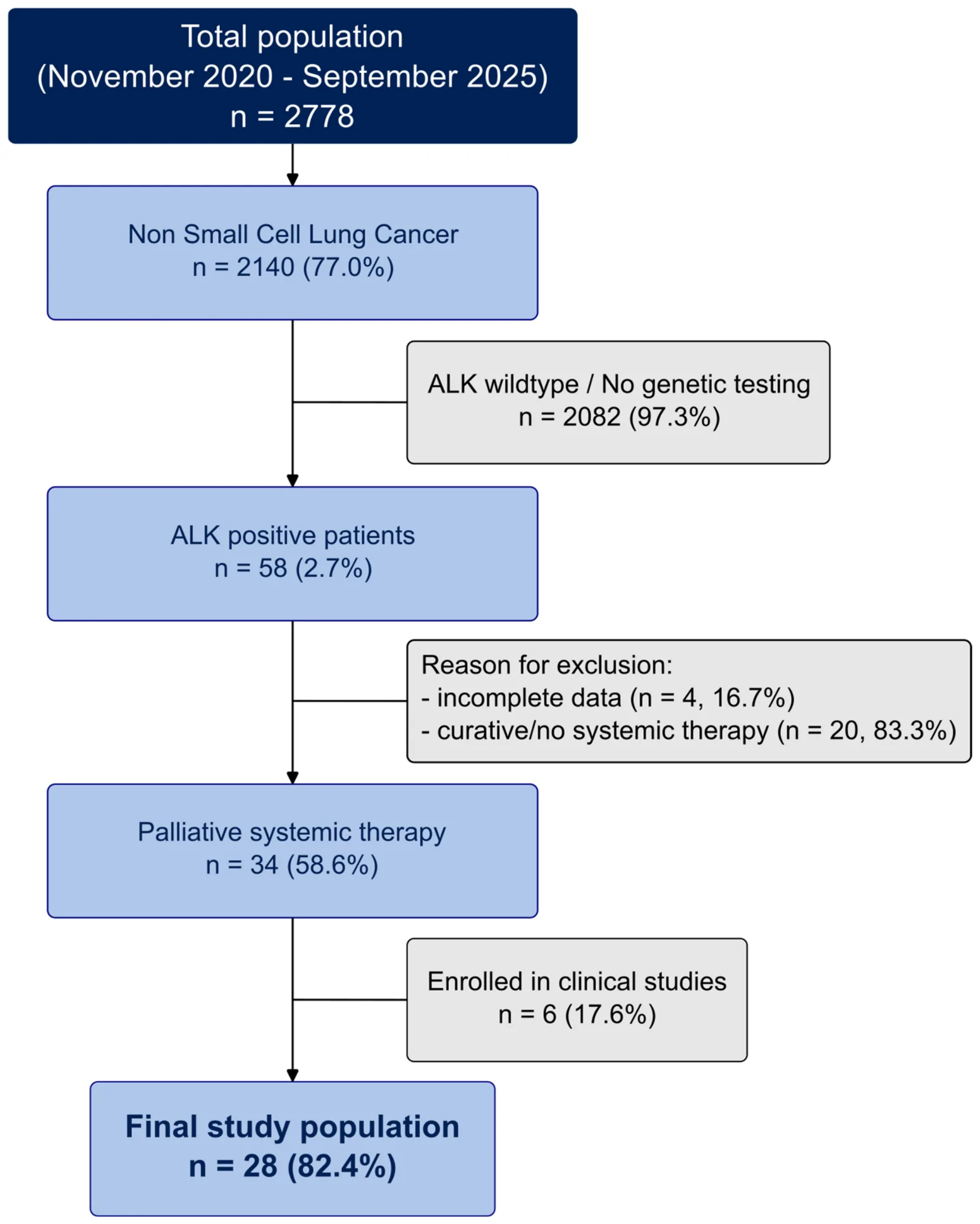
Patient selection flowchart.

From this overall cohort, patients with ALK-rearranged NSCLC represent a small molecularly defined subgroup consistent with the known low prevalence of ALK alterations. For the present analysis, only patients with confirmed ALK rearrangement detected by next-generation sequencing and treated with palliative intent were included. Patients with early-stage disease treated with curative intent and/or without systemic therapy (*n* = 20), or with incomplete treatment data (*n* = 4), were excluded. This resulted in a final study population of a total of 28 patients with ALK-positive lung cancer; 64.3% were alive at the time of data cut-off (30 June 2026). A detailed patient selection flowchart can be found in Figure 1. Sex distribution was nearly balanced, with 53.6% men and 46.4% women. The median age at diagnosis was 54 years (range: 26–84).

Half of the patients (50.0%) were never smokers, 32.1% were former smokers, and 14.3% were current smokers. The median smoking exposure among former and current smokers was 10 pack-years (range: 2–40), with 53.8% reporting between 1 and 10 pack-years.

Among the 28 patients with available ALK fusion variant data, EML4-ALK variant 1 was the most frequent rearrangement, identified in 19 patients (67.9%). EML4-ALK variant 3 was detected in five patients (17.9%), while EML4-ALK variant 2 was observed in two patients (7.1%). In an additional two patients (7.1%), the specific ALK rearrangement variant was unknown.

Most patients presented with an excellent performance status: 64.3% had an Eastern Cooperative Oncology Group (ECOG) performance status of 0, 17.9% had ECOG 1, and 14.3% had ECOG 2. At initial diagnosis, advanced disease stages predominated, with 32.1% of patients in stage IVA and 53.6% in stage IVB. Tumor stage IIIB was reported in 7.1% of patients while tumor stages IIB and IIIA were each observed in one patient (3.6%). These patients were treated with palliative intent, as local curative treatment approaches were not considered feasible due to comorbidities and impaired performance status. Histologically, adenocarcinoma was the most common subtype, accounting for 92.9% of cases. Patient characteristics are summarized in Table 1.

### 3.2. Treatment Lines and Selection of ALK Inhibitors

#### 3.2.1. Treatment Lines

Overall, 49 lines of systemic therapy were administered to 28 patients included in this analysis. At data cut-off, 65.3% (*n* = 32) of treatment lines had ended, while 34.7% (*n* = 17) were still ongoing. Systemic therapies included second-generation ALK inhibitors (alectinib and brigatinib), the third-generation ALK inhibitor lorlatinib, and chemotherapy and chemoimmunotherapy.

Of 28 patients, 26 received targeted therapy in the first-line setting, while two patients received chemoimmunotherapy due to missing NGS results at the time of treatment decision. Nine patients proceeded to second-line treatment, all but one of whom were treated exclusively with ALK inhibitors. The one patient without ALK inhibitor treatment received chemotherapy. Seven patients received third-line therapy: four were treated with ALK inhibitors (one in combination with chemotherapy), while two received chemoimmunotherapy and one patient received immunotherapy alone. Three patients reached fourth-line therapy, with two treated with an ALK inhibitor and one with chemotherapy. Fifth-line therapy was documented in two patients; one of whom received an ALK inhibitor and one received chemotherapy. Reasons for not receiving an ALK inhibitor were the lack of NGS results at the time of treatment decision in all three patients receiving first- or second-line therapy. In the third-line setting, all three patients not receiving an ALK inhibitor had experienced disease progression under ALK inhibitor treatment. In the fourth- and fifth-line settings, no further ALK inhibitor option was available for the two patients concerned.

**Figure 2:**
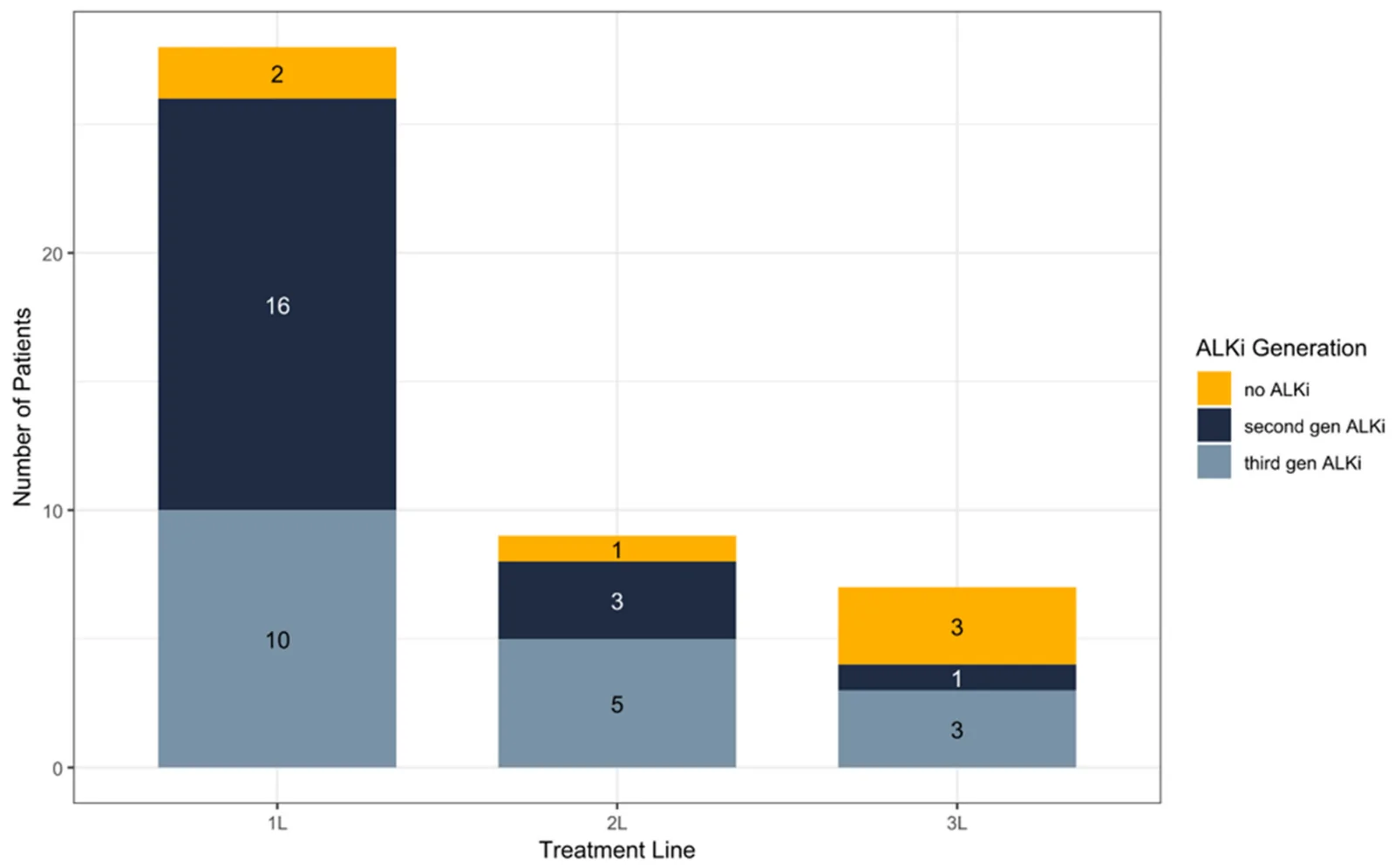
Real-world treatment sequencing in patients with ALK-positive NSCLC treated with palliative intent.

Treatment lines up to third-line therapy are illustrated in Figure 2.

#### 3.2.2. Selection of ALK Inhibitor Generation

##### Selection of ALK Inhibitor Generation by Treatment Lines

Across all 41 ALK treatment lines, 53.7% (*n* = 22) of ALK inhibitor treatments consisted of second-generation ALK inhibitors, while 46.3% (*n* = 19) involved third-generation ALK inhibitors. Treatment had ended in 16 second-generation ALK inhibitor treatment lines and in nine third-generation ALK inhibitor treatment lines. At the time of analysis, six treatment lines remained ongoing with second-generation ALK inhibitors and in ten third-generation ALK inhibitors treatment lines. However, while lorlatinib had been available for later-line treatment since May 2019, it was approved for first-line treatment of ALK-positive advanced NSCLC in January 2022. Therefore, comparing the use of third-generation ALK inhibitors between 2019–2021 and 2022 and onwards allows for an assessment of changes in the use of different generations of ALK inhibitors which can be found in Table 2.

##### Selection of ALK Inhibitor Generation by Patient

In the first-line setting, 16 patients received second-generation ALK inhibitors (nine alectinib, seven brigatinib), while ten patients received the third-generation ALK inhibitor lorlatinib. In second-line therapy, three patients were treated with brigatinib and five patients with lorlatinib. In the third-line setting, one patient received brigatinib and three patients received lorlatinib. In the fourth-line setting, one patient was treated with brigatinib and one patient with lorlatinib.

##### Selection of ALK Inhibitor Generation in Patients with Brain Metastasis

In this study population, four patients had metastasis of the brain. In the first-line setting, three patients received second-generation therapy, and one patient was treated with third-generation ALK inhibitors. One of the four patients with brain metastasis had second-line treatment with a third-generation ALK inhibitor followed by a third-line treatment with a combination of third-generation ALK inhibitor, Pemetrexed and Carboplatin. One patient received Gamma Knife radiosurgery, whereas the other three did not receive brain-directed radiotherapy. Initially, three patients achieved a complete intracranial response (CR; 75%) and one a partial response (PR; 25%). Subsequently, three patients experienced disease progression, while one remains on ongoing treatment with a second-generation ALK inhibitor.

### 3.3. Treatment Patterns

#### 3.3.1. Median Duration of Treatment

At data cut-off, ALK inhibitor treatment was ongoing in 16 of 41 treatment lines (39.0%), thus duration of treatment (DOT) was further evaluated using Kaplan–Meier estimates. Overall, the estimated median DOT was 287 days (95% CI, 172-NE) for second-generation ALK inhibitor and 736 days (95% CI, 376-NE) for third-generation ALK inhibitor.

In the first-line setting, the estimated median DOT was 636.5 days (95% CI, 172-NE) for second-generation ALK inhibitors, whereas the median was not reached for lorlatinib (95% CI, 736-NE). Among individual second-generation ALK inhibitors used in the first-line setting, the estimated median DOT was 497 days (95% CI, 152-NE) for alectinib and 1301 days (95% CI, 172-NE) for brigatinib.

In the second-line setting, the estimated median DOT was 92 days (95% CI, 19-NE) for brigatinib and 376 days (95% CI, 51-NE) for lorlatinib. In the third- to fifth-line setting, the estimated median DOT was 181 days (95% CI, 48-NE) for second-generation brigatinib and alectinib and 63 days (95% CI, 51-NE) for third-generation lorlatinib.

Among discontinued treatment lines, the observed median DOT was 176.5 days (range, 19–1301) for second-generation ALK inhibitors and 123.0 days (range, 34–736) for third-generation ALK inhibitors. In the first-line setting, the observed median DOT among discontinued treatments lines was 260.5 days (range, 55–1301) for second-generation ALK inhibitors and 517.0 days (range, 298–736) for lorlatinib. Detailed information on estimated DOT and DOT of discontinued treatment is shown in Table 3.

#### 3.3.2. Treatment Discontinuation

Overall, 25 ALK inhibitor treatment lines were discontinued. The main reasons for discontinuation in second- and third-generation ALK inhibitor were progressive disease with 11/16 (68.8%) and 7/9 (77.8%) treatment lines, respectively. Death was the reason for discontinuation in two treatment lines each (2/16 12.5%, 2/9 22.2%) in the second- and third-generation ALK inhibitor. One patient received treatment beyond progression. Adverse events (AEs)—including pneumonitis G2, pneumonitis G3 and skin and subcutaneous tissue disorders G2—led to treatment discontinuation in 3/16 treatment lines (18.8%) with second-generation ALK inhibitor treatment.

### 3.4. Efficacy Analysis

**Figure 3:**
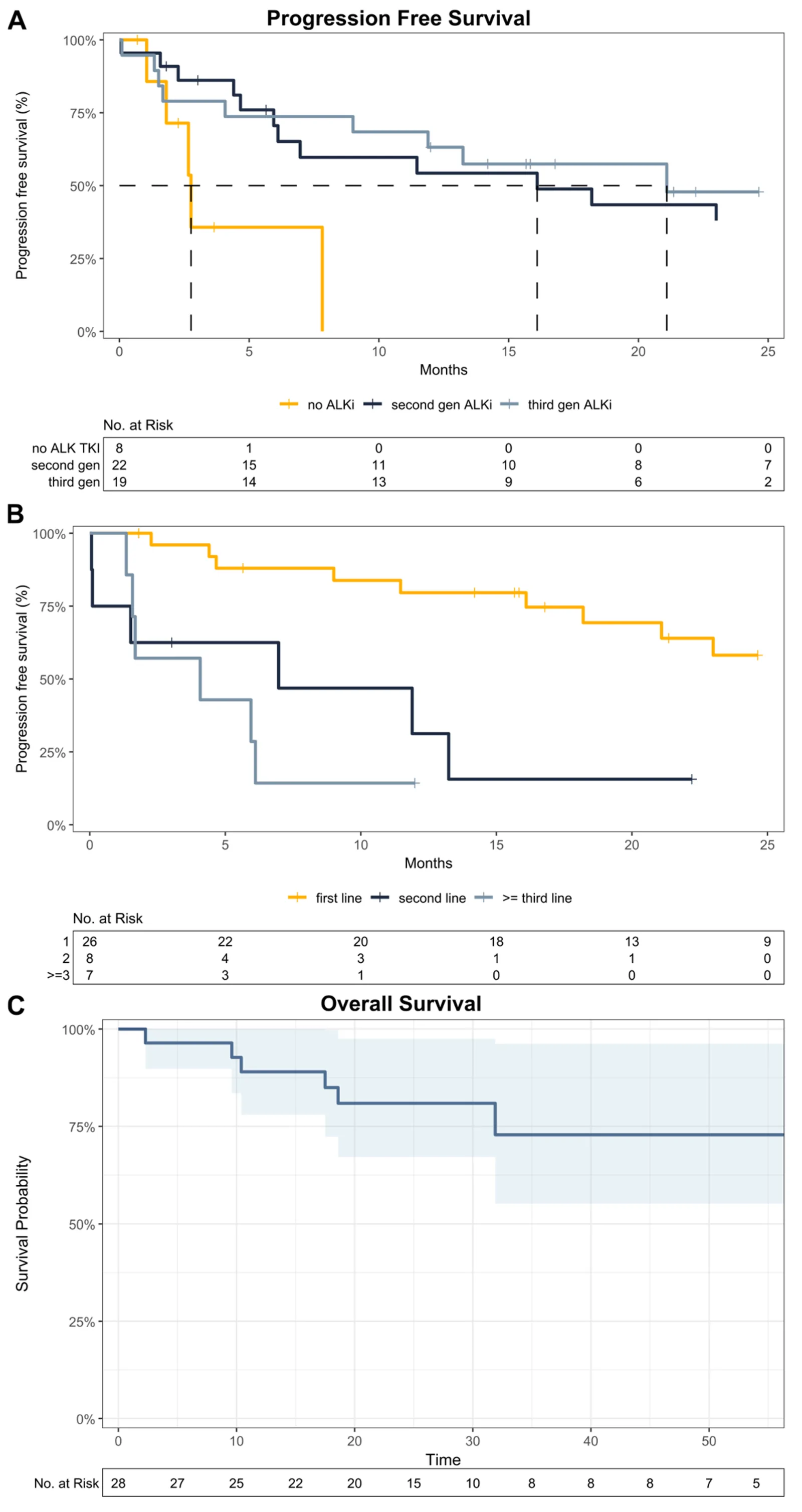
Kaplan–Meier curves and corresponding risk tables are displayed until the number at risk decreases below five. (**A**) Kaplan–Meier estimates of progression-free survival according to treatment type. (**B**) Kaplan–Meier estimates of progression-free survival according to ALK inhibitor treatment line. (**C**) Kaplan–Meier estimates of overall survival for the study population.

Across all treatment lines, the ORR was 77.3% (*n* = 17/22) (95% CI, 55–92) in second-generation ALK inhibitor treatment and 73.7% (*n* = 14/19) (95% CI, 49–91) in third-generation ALK inhibitor treatment, indicating that more than two-thirds of treatment lines achieved complete or partial response under ALK inhibitor treatment. In total, 11 CR and 20 PR were documented. SD and PD were observed in four treatment lines each. One treatment line was not evaluable, and best response was unknown for one treatment line. Detailed information on antitumor activity according to treatment line can be found in Table 4. Median progression-free survival (PFS) during ALK inhibitor treatment overall was 18.2 months (95% CI, 11.5-NE). In patients receiving treatment with second-generation and third-generation ALK inhibitor, PFS was 16.1 (95% CI, 6.1-NE) and 21.1 (95% CI, 11.9-NE), respectively (Figure 3A). Patients without ALK inhibitor treatment showed a PFS of 2.8 months (95% CI, 1.8-NE). Median PFS was 42.7 months (95% CI, 18.2-NE) in the first-line setting, 9.4 months (95% CI, 1.5-NE) in the second-line setting, and 2.7 months (95% CI, 1.7-NE) in the third or later lines (Figure 3B).

Overall survival was not reached due to the short period of observation (Figure 3C). Survival at 12 months, 24 months and 36 months was 89% (95% CI, 78–100%) 81% (95% CI, 67–98%) and 73% (95% CI, 55–96%), respectively.

### 3.5. Other Systemic Treatments

Beyond ALK inhibitor therapy, two patients initially received first-line pembrolizumab, pemetrexed, and carboplatin because the initial histopathological diagnosis was NSCLC-NOS or large-cell carcinoma. As the immunohistochemical findings were considered inconclusive, systemic treatment was initiated while awaiting confirmation of the molecular profile by next-generation sequencing (NGS). In the second-line setting, one patient received carboplatin and gemcitabine treatment. In the third-line setting, one patient received immunotherapy with pembrolizumab and two patients were treated with chemoimmunotherapy (carboplatin, pemetrexed, and pembrolizumab). In the fourth-line setting, one patient received pemetrexed and carboplatin, while in the fifth-line setting, one patient was treated with docetaxel.

### 3.6. Radiation Therapy by Patient

Within this study cohort, 17 patients (60.7%) received radiation therapy. Fourteen of these patients (82.4%) were treated with radiation therapy during and three patients (17.6%) after ALK inhibitor treatment. Nine patients (52.9%) had oligometastatic radiotherapy. The targets were predominantly oligometastasis of the spine (*n* = 5, 55.6%) and brain (*n* = 3, 33.3%).

### 3.7. Adverse Events

Across all 41 lines of ALK inhibitor treatment, adverse events (AEs) of any grade and grade ≥ 3 were reported in 30 (73.2%) lines and in 5 lines (12.2%), respectively (Supplementary Table S1). AEs led to dose reductions in 14 treatment lines (34.1%), AE-induced treatment holidays in 11 treatment lines (26.8%) and discontinuations in 3 treatment lines (7.3%). The most common AEs in second-generation ALK inhibitors were edema of the limbs, increased CPK, and eczema (each two events; 9.1% overall). Edema of the limbs was most frequent with alectinib (two events, 20.0%), whereas increased CPK and eczema were most frequent with brigatinib (two events, 16.7% each). The only observed grade-3 AEs in second-generation ALK inhibitors were pneumonitis under alectinib treatment (one event, 10.0%), and pneumonia and pneumonitis under brigatinib treatment (one event each, 8.3%). In treatment with the third-generation ALK inhibitor lorlatinib, hypercholesterolemia was the predominant AE, occurring in seven treatment lines (36.8%), followed by edema of the limbs (four events, 21.1%). Grade-3 AEs in third-generation ALK inhibitor lorlatinib included elevated liver enzymes (ALT and AST) and cognitive disturbance, each occurring in one treatment line (5.3%). Details of all reported adverse events can be found in Supplementary Table S1.

### 3.8. Dose Reductions

Dose reductions were documented in 14 treatment lines (34.1%). Among second-generation ALK inhibitor, dose reductions occurred in five treatment lines (22.7%) with a median TTDR of 65 days (range: 42–181), while nine treatment lines (47.4%) with third-generation ALK inhibitor required dose reductions with a median TTDR of 95 days (range: 9–654).

For second-generation ALK inhibitor, dose reductions occurred in four first-line treatment lines and in one third-line treatment. For third-generation ALK inhibitors, dose reductions were required in five first-line treatment lines and in two second- and one third- and fourth-line treatment. Specifically, the standard dose of alectinib (2 × 600 mg daily) and brigatinib (180 mg daily) was reduced in two first-line therapy lines with a median TTDR of 53.5 days (range: 42–65) and 115.0 days (range: 49–181), respectively. In the third-line setting, brigatinib (180 mg daily) required dose reductions in one treatment line with a TTDR of 74 days. Lorlatinib (100 mg daily) was reduced in five first-line treatment lines (median TTDR = 114 (range: 26–249)) and in two second-line treatments (median TTDR = 374.5 (range: 95–654)) and in one treatment line each in third- (TTDR = 60 days) and fourth-line treatment (TTDR = 9 days). All treatment reductions occurred due to adverse events, the details of which can be found according to ALK inhibitor therapy in Supplementary Table S1.

### 3.9. Treatment Holidays

Treatment holidays were documented in 13 treatment lines (31.7%) with ALK inhibitors, including 11 treatment holidays (84.6%) due to AEs. Six second-generation (6/22; 27.3%) and seven third-generation ALK inhibitor treatment lines (7/19; 36.8%) were temporarily suspended. Treatment holidays occurred in second-generation ALK inhibitors during first-line therapy in four treatment lines (4/16; 25.0%; (two lines (2/9; 22.2%) with alectinib and two (2/7; 28.6%) with brigatinib) and in two second-line brigatinib treatment lines (2/3; 66.7%). Under lorlatinib treatment, four treatment holidays (4/10; 40.0%) occurred during first-line therapy and one each during second-, third- and fourth-line therapy (1/5, 1/3, 1/1). Median time-to-treatment-holiday was 44 days (range: 27–61) and 223 days (range: 118–1294) in second-generation ALK inhibitor alectinib and brigatinib, respectively. Third-generation ALK inhibitor lorlatinib reported a median time-to-treatment-holiday of 88 days (range: 9–147). Reasons for treatment holidays accounted mainly for adverse events, except in two cases of patients’ decisions: in one case, treatment was suspended for 9 days and in another case, for 60 days. Details of all treatment holidays due to adverse events can be found in Supplementary Table S1.

## 4. Discussion

The primary contribution of the present study is the characterization of contemporary real-world treatment pathways in patients with ALK-positive NSCLC treated within a multicenter registry. Unlike randomized clinical trials, this cohort captures routine clinical decision-making, sequential use of multiple ALK inhibitors, treatment modifications, temporary interruptions, and continuation of targeted therapy beyond radiological progression in a heterogeneous patient population. The treatment landscape of ALK-rearranged advanced NSCLC has undergone substantial evolution over the past decade. Second- and third-generation ALK tyrosine kinase inhibitors (TKIs) have demonstrated superior efficacy compared with first-generation crizotinib in randomized clinical trials, particularly with respect to progression-free survival and intracranial disease control, establishing next-generation ALK inhibitors as preferred first-line treatment options [5,6,7,8,9,16,17].

However, whether outcomes observed in randomized clinical trials translate into routine clinical practice remains an important question, because patients treated outside clinical trials are generally more heterogeneous and may differ with respect to age, comorbidities, performance status, and prior treatment exposure. Real-world studies have therefore provided complementary information on treatment sequencing, treatment duration, and treatment modifications in routine practice [11,12,13,14]. Treatment patterns in our cohort reflected the evolution of evidence-based management of ALK-positive NSCLC. Second-generation ALK inhibitors remained the most frequently used agents overall, while lorlatinib was increasingly adopted during the later study period in both first- and later-line settings. The increasing use of lorlatinib in our cohort likely reflects evolving clinical evidence, updated guideline recommendations, and increasing availability during the study period rather than differences in patient characteristics alone [16,17,18,19]. Although patients receiving third-generation ALK inhibition had a descriptively longer estimated duration of treatment within this registry cohort, these findings should be interpreted as observational treatment patterns rather than evidence of superior efficacy. The study was not designed or powered to compare outcomes between ALK inhibitor generations, and treatment groups differed with respect to calendar period, follow-up duration, and prior treatment exposure. Furthermore, confidence intervals were wide and substantially overlapped because of the limited sample size and the high proportion of ongoing treatments at the data cut-off. Consequently, observed treatment duration should not be interpreted as a head-to-head comparison between second- and third-generation ALK inhibitors.

Notably, the median first-line duration of treatment was not reached for lorlatinib at the time of data cut-off. Given the limited number of patients and the short follow-up, this observation should be interpreted as descriptive only and requires confirmation in larger cohorts with longer follow-up. Objective response rates exceeded 70% for both second- and third-generation ALK inhibitors in our cohort, consistent with the substantial antitumor activity observed with next-generation ALK inhibitors in prospective clinical trials [5,6,7]. Similar treatment activity has also been reported in real-world cohorts [12,14]. Direct comparisons should nevertheless be interpreted cautiously because our cohort included different therapy lines, heterogeneous prior treatment exposure, and limited patient numbers. The relatively short median PFS should also be interpreted cautiously because patients were treated across different therapy lines and had heterogeneous prior treatment exposure. In addition, treatment-line analyses may be influenced by treatment selection and calendar-time effects inherent to an observational registry.

Adverse events were frequent but generally manageable. Although almost three quarters of treatment lines were associated with at least one adverse event, grade ≥3 toxicities were uncommon and treatment discontinuation due to toxicity occurred only rarely. Dose reductions and temporary treatment interruptions were frequently employed, allowing for continuation of targeted therapy in most patients. These findings are consistent with the toxicity profiles reported in prospective studies [5,6,7,12,14] and emphasize the importance of proactive adverse-event management in maintaining long-term ALK inhibition. Continuous ALK inhibition may be particularly relevant in patients with central nervous system (CNS) involvement, which represents a clinically important manifestation of ALK-positive NSCLC [20]. Next-generation ALK inhibitors have demonstrated substantially improved intracranial activity compared with crizotinib. Alectinib and brigatinib demonstrated superior intracranial activity compared with crizotinib [8,9], while lorlatinib showed particularly pronounced and durable intracranial efficacy in the CROWN study [16,18,21]. These findings underscore the clinical importance of effective intracranial disease control and sustained ALK inhibition in patients with ALK-positive NSCLC [8,9,19]. The presence of CNS metastases may therefore influence treatment selection in routine clinical practice, given the established intracranial activity of next-generation ALK inhibitors [8,9,16,21]. Patients with brain metastases were generally treated with next-generation ALK inhibitors whenever clinically appropriate, consistent with the established intracranial efficacy of these agents [8,9,16,21]. However, only four patients in our cohort presented with brain metastases, precluding any meaningful subgroup analysis of CNS outcomes. These observations should therefore be interpreted descriptively.

In patients with oligoprogressive disease, local ablative therapy can permit continuation of the same targeted therapy and may provide additional disease control [22]. In addition, continuation of lorlatinib beyond radiological progression has been described in selected patients, particularly when ongoing clinical benefit is maintained despite limited progression [23]. These approaches are particularly relevant in oncogene-driven NSCLC, in which progression may be limited to a small number of sites while systemic disease remains controlled [22,23]. When targeted treatment options were exhausted or additional resistance mechanisms emerged, patients were treated with platinum–pemetrexed-based chemotherapy or, in selected cases, additional targeted therapies, according to individual clinical circumstances. This treatment pattern is consistent with current guideline recommendations supporting sequential use of available ALK inhibitors whenever clinically appropriate, followed by platinum-based chemotherapy after exhaustion of targeted treatment options [17,24]. Platinum–pemetrexed-based chemotherapy has also demonstrated activity in previously treated ALK-positive NSCLC [25].

Lorlatinib has demonstrated clinically meaningful and durable activity in patients previously treated with one or more ALK inhibitors, supporting its role within sequential treatment strategies [26,27]. At the same time, sequential exposure to increasingly potent ALK inhibitors may select for complex ALK resistance mutations, highlighting the biological challenges associated with prolonged sequential ALK inhibition [28,29].

Several limitations should be acknowledged. First, although patients were prospectively enrolled and followed within the LALUCA registry [15], the present study represents a retrospective analysis of prospectively collected registry data. The relatively small sample size limited statistical power and precluded formal comparative analyses between individual ALK inhibitors. Second, some analyses were performed at the treatment-line level, allowing individual patients to contribute multiple treatment lines over the course of their disease. Consequently, treatment-line observations are not statistically independent and should be interpreted as descriptive measures of real-world treatment exposure rather than patient-level comparative outcomes. Third, treatment allocation reflected evolving standards of care and an increasing availability of next-generation ALK inhibitors over the study period, introducing potential calendar-time and selection bias. Fourth, survival analyses remain immature because a considerable proportion of treatment lines were still receiving treatment at the data cut-off, resulting in wide confidence intervals for treatment duration estimates. Finally, molecular resistance mechanisms at progression were not systematically assessed and therefore could not be correlated with treatment sequencing. Accordingly, the findings of this observational study should be interpreted as descriptive and hypothesis-generating rather than evidence of comparative treatment effectiveness.

## 5. Conclusions

This study provides descriptive real-world evidence on contemporary treatment sequencing in ALK-positive NSCLC from a retrospective analysis of a prospective multicenter registry. The prospective design of the underlying LALUCA registry, together with detailed documentation of treatment modifications and sequential ALK inhibitor use, represents a key strength of the study. As therapeutic options continue to evolve, larger collaborative registries with longer follow-up will be important to characterize real-world treatment sequencing, resistance patterns, CNS outcomes, and the long-term clinical benefit associated with next-generation ALK inhibition.

## Figures and Tables

**Table 1:** Baseline patient characteristics for ALK-positive lung cancer patients with palliative treatment intent.

Characteristics at Diagnosis	All Patients (*n* = 28)
Age, years	
Median (range)	54 (26–84)
Age groups, *n* (%)	
<=40 years	4 (14.3)
41–50 years	8 (28.6)
51–60 years	7 (25.0)
61–70 years	4 (14.3)
71–80 years	3 (10.7)
>80 years	2 (7.1)
Gender, *n* (%)	
Male	15 (53.6)
Female	13 (46.4)
Histology, *n* (%)	
Adenocarcinoma	26 (92.9)
NSCLC/NOS/LC	2 (7.1)
Smoking status, *n* (%)	
Never smoker	14 (50.0)
Former smoker	9 (32.1)
Current smoker	4 (14.3)
Unknown	1 (3.6)
Pack years, *n* (%)	
Smoker (≤30 py)	12 (92.3)
Heavy smoker (>30 py)	1 (7.7)
ECOG performance status, *n* (%)	
ECOG PS 0	18 (64.3)
ECOG PS 1	5 (17.9)
ECOG PS 2	4 (14.3)
Unknown	1 (3.6)
Metastases at diagnosis, *n* (%) *	*n* = 24
Bone	9 (37.5)
Pleural effusion	9 (37.5)
Liver	6 (25.0)
Distant lymph node	5 (20.8)
Brain	4 (16.7)
Adrenal gland	1 (4.2)
Other	7 (29.2)
ALK rearrangements	*n* = 28
EML4-ALK V1 (E13:A20)	19
EML4-ALK V3 (E6:A20)	5
EML4-ALK V2 (E20:A20)	2
Unknown ALK rearrangement	2

NSCLC—non-small cell lung cancer; NOS—NSCLC not otherwise specified; LC—large-cell lung carcinoma; ECOG PS—Eastern Cooperative Oncology Group performance status; * percentages are only calculated for patients with advanced tumor stage.

**Table 2:** ALK inhibitor generation according to year of treatment start.

Line of Therapy	Year of Treatment Start	ALK Inhibitor Generation	*n*	% *
First-line	2019–2021	Second gen	6	100.0
	≥2022	Second gen	10	50.0
		Third gen	10	50.0
Second-line	2019–2021	Second gen	1	100.0
	≥2022	Second gen	2	28.6
		Third gen	5	71.4
Third-line	2019–2021	Third gen	1	100.0
	≥2022	Second gen	1	33.3
		Third gen	2	66.7
Fourth-line	≥2022	Second gen	1	50.0
		Third gen	1	50.0
Fifth-line	2019–2021	Second gen	1	100.0

* Percentages are based on the total number of patients within each line of therapy and treatment period subgroup; therefore, percentages sum to 100% within each treatment line and calendar-period category.

**Table 3:** Kaplan–Meier estimates of median treatment duration and observed duration among discontinued treatments by ALK inhibitor generation and line of therapy.

Line of Therapy	ALK Inhibitor Generation	Drug	*n*	Median Duration (Days)	95% CI	Discontinued Treatments *	
						*n*	Median (Range)
Overall	Second gen	All	22	287	172-NE	16	176.5 (19–1301)
	Third gen	All	19	736	376-NE	9	123.0 (34–736)
First-line (*n* = 26)	Second gen	All	16	636.5	172-NE	10	260.5 (55–1301)
		Alectinib	9	497	152-NE	6	250.5 (55–559)
		Brigatinib	7	1301	172-NE	4	443.0 (69–1301)
	Third gen	Lorlatinib	10	NE	736-NE	2	517.0 (298–736)
Second-line (*n* = 8)	Second gen	Brigatinib	3	92	19-NE	3	92.0 (19–225)
	Third gen	Lorlatinib	5	376	51-NE	4	213.5 (34–403)
≥Third-line (*n* = 7)	Second gen	Alectinib/Brigatinib	3	181	48-NE	3	181.0 (48–192)
	Third gen	Lorlatinib	4	63	51-NE	3	63.0 (51–123)

Median duration of treatment was estimated using the Kaplan–Meier method. Patients remaining on treatment at the data cut-off date (30 June 2026) were censored at that date. NE = not estimable. * Observed treatment duration among discontinued treatment lines only, reported as median (range) in days and not derived from Kaplan–Meier estimates.

**Table 4:** Antitumor activity.

Antitumor Activity	1L	2L	≥3L
Second-generation ALK inhibitor	*n* = 16	*n* = 3	*n* = 3
ORR, *n* (%)	14 (87.5)	1 (33.3)	2 (66.7)
95% CI	61.7–98.4	0.8–90.6	9.4–99.2
CR, *n* (%)	5 (31.3)	0	0
PR, *n* (%)	9 (56.3)	1 (33.3)	2 (66.7)
SD, *n* (%)	1 (6.3)	0	0
PD, *n* (%)	0	2 (66.7)	0
NE, *n* (%)	0	0	1 (33.3)
Unknown, *n* (%)	1 (6.3)	0	0
Third-generation ALK inhibitor	*n* = 10	*n* = 5	*n* = 4
ORR, *n* (%)	9 (90.0)	2 (40.0)	3 (75.0)
95% CI	55.5–99.7	5.3–85.3	19.4–99.4
CR, *n* (%)	5 (50.0)	0	1 (25.0)
PR, *n* (%)	4 (40.0)	2 (40.0)	2 (50.0)
SD, *n* (%)	1 (10.0)	2 (40.0)	0
PD, *n* (%)	0	1 (20.0)	1 (25.0)
NE, *n* (%)	0	0	0
Unknown, *n* (%)	0	0	0

Due to rounding, percentages may not add up to 100%.

## Data Availability

The original contributions presented in this study are included in the article/Supplementary Material. Further inquiries can be directed to the corresponding author.

## Supplementary Materials

The following supporting information can be downloaded at https://www.mdpi.com/article/10.3390/cancers18213470/s1: Table S1: Adverse Events.
