# Supplementary material for: Real-World Treatment Patterns and Sequencing of ALK-Inhibitors in ALK-Positive NSCLC Treated with Palliative Intent
Source: Cancers (Basel). 2026 Oct 27;18(21):3470. doi: 10.3390/cancers18213470 (PMC13762226; doi:10.3390/cancers18213470)
Supplement: Supplementary file 1 [file cancers-18-03470-s001.zip › cancers-4634579-supplementary.pdf]

Supplementary material

Table S1. Adverse Events

Adverse Events	Any Grade	G 1	G 2	G 3	G 4	Dose reduct.	Treatment holiday	Treatment discont.
**Second-generation ALK inhibitor**								
**Alectinib (n=10)**								
Alanine aminotransferase increased	1 (10.0)	0 (0.0)	1 (10.0)	0 (0.0)	0 (0.0)	1 (10.0)	1 (10.0)	0 (0.0)
Aspartate aminotransferase increased	1 (10.0)	0 (0.0)	1 (10.0)	0 (0.0)	0 (0.0)	1 (10.0)	1 (10.0)	0 (0.0)
Creatinine increased	1 (10.0)	1 (10.0)	0 (0.0)	0 (0.0)	0 (0.0)	1 (10.0)	1 (10.0)	0 (0.0)
Dyspnea	1 (10.0)	0 (0.0)	1 (10.0)	0 (0.0)	0 (0.0)	0 (0.0)	0 (0.0)	0 (0.0)
Edema limbs	2 (20.0)	1 (10.0)	1 (10.0)	0 (0.0)	0 (0.0)	0 (0.0)	1 (10.0)	0 (0.0)
Platelet count decreased	1 (10.0)	1 (10.0)	0 (0.0)	0 (0.0)	0 (0.0)	1 (10.0)	1 (10.0)	0 (0.0)
Pleural effusion	1 (10.0)	0 (0.0)	1 (10.0)	0 (0.0)	0 (0.0)	0 (0.0)	0 (0.0)	0 (0.0)
Pneumonitis	1 (10.0)	0 (0.0)	0 (0.0)	1 (10.0)	0 (0.0)	0 (0.0)	0 (0.0)	1 (10.0)
**Brigatinib (n=12)**								
Alanine aminotransferase increased	1 (8.3)	0 (0.0)	1 (8.3)	0 (0.0)	0 (0.0)	1 (8.3)	1 (8.3)	0 (0.0)
Aspartate aminotransferase increased	1 (8.3)	1 (8.3)	0 (0.0)	0 (0.0)	0 (0.0)	1 (8.3)	1 (8.3)	0 (0.0)
CPK increased	2 (16.7)	1 (8.3)	0 (0.0)	0 (0.0)	0 (0.0)	1 (8.3)	1 (8.3)	0 (0.0)
Creatinine increased	1 (8.3)	0 (0.0)	1 (8.3)	0 (0.0)	0 (0.0)	0 (0.0)	0 (0.0)	0 (0.0)
Dyspnea	1 (8.3)	1 (8.3)	0 (0.0)	0 (0.0)	0 (0.0)	0 (0.0)	0 (0.0)	0 (0.0)

Eczema	2 (16.7)	0 (0.0)	2 (16.7)	0 (0.0)	0 (0.0)	1 (8.3)	0 (0.0)	0 (0.0)
Nausea	1 (8.3)	1 (8.3)	0 (0.0)	0 (0.0)	0 (0.0)	0 (0.0)	0 (0.0)	0 (0.0)
Pneumonia	1 (8.3)	0 (0.0)	0 (0.0)	1 (8.3)	0 (0.0)	0 (0.0)	0 (0.0)	0 (0.0)
Pneumonitiis	1 (8.3)	0 (0.0)	0 (0.0)	1 (8.3)	0 (0.0)	0 (0.0)	1 (8.3)	1 (8.3)
Skin and subcutaneous tissue disorders	1 (8.3)	0 (0.0)	1 (8.3)	0 (0.0)	0 (0.0)	0 (0.0)	0 (0.0)	1 (8.3)
**Lorlatinib (n=19)**								
Alanine aminotransferase increased	3 (15.8)	0 (0.0)	2 (10.5)	1 (5.3)	0 (0.0)	2 (10.5)	2 (10.5)	0 (0.0)
Aspartate aminotransferase increased	3 (15.8)	1 (5.3)	1 (5.3)	1 (5.3)	0 (0.0)	2 (10.5)	2 (10.5)	0 (0.0)
Blood lactate dehydrogenase increased	1 (5.3)	1 (5.3)	0 (0.0)	0 (0.0)	0 (0.0)	1 (5.3)	0 (0.0)	0 (0.0)
Cholesterol high	7 (36.8)	2 (10.5)	5 (26.3)	0 (0.0)	0 (0.0)	1 (5.3)	0 (0.0)	0 (0.0)
Cognitive disturbance	1 (5.3)	0 (0.0)	0 (0.0)	1 (5.3)	0 (0.0)	1 (5.3)	1 (5.3)	0 (0.0)
Concentration impairment	1 (5.3)	0 (0.0)	1 (5.3)	0 (0.0)	0 (0.0)	1 (5.3)	1 (5.3)	0 (0.0)
Delirium	1 (5.3)	0 (0.0)	1 (5.3)	0 (0.0)	0 (0.0)	1 (5.3)	0 (0.0)	0 (0.0)
Edema limbs	4 (21.1)	2 (10.5)	2 (10.5)	0 (0.0)	0 (0.0)	1 (5.3)	2 (10.5)	0 (0.0)
Fever	1 (5.3)	1 (5.3)	0 (0.0)	0 (0.0)	0 (0.0)	0 (0.0)	0 (0.0)	0 (0.0)
Headache	1 (5.3)	0 (0.0)	1 (5.3)	0 (0.0)	0 (0.0)	1 (5.3)	0 (0.0)	0 (0.0)
Hyperlipidemia	3 (15.8)	3 (15.8)	0 (0.0)	0 (0.0)	0 (0.0)	1 (5.3)	0 (0.0)	0 (0.0)
Hypertriglyceridemia	3 (15.8)	3 (15.8)	0 (0.0)	0 (0.0)	0 (0.0)	0 (0.0)	0 (0.0)	0 (0.0)
Lipase increased	1 (5.3)	0 (0.0)	1 (5.3)	0 (0.0)	0 (0.0)	1 (5.3)	0 (0.0)	0 (0.0)
Peripheral sensory neuropathy	1 (5.3)	1 (5.3)	0 (0.0)	0 (0.0)	0 (0.0)	0 (0.0)	0 (0.0)	0 (0.0)

Tinnitus	1 (5.3)	1 (5.3)	0 (0.0)	0 (0.0)	0 (0.0)	0 (0.0)	1 (5.3)	0 (0.0)

Adverse events (AEs) observed during treatment with the ALK inhibitors alectinib, brigatinib, and lorlatinib. Of a total of 49 treatment lines, ALK inhibitors were administered in 41 (83.7%). Only AEs occurring during ALK inhibitor therapy are reported.
